# Pathogenic cryptic variants detectable through exome data reanalysis significantly increase the diagnostic yield in Joubert syndrome

**DOI:** 10.1038/s41431-024-01703-x

**Published:** 2024-10-11

**Authors:** Fulvio D’Abrusco, Valentina Serpieri, Cecilia Maria Taccagni, Jessica Garau, Luca Cattaneo, Monica Boggioni, Simone Gana, Roberta Battini, Enrico Bertini, Ginevra Zanni, Eugen Boltshauser, Renato Borgatti, Romina Romaniello, Sabrina Signorini, Vincenzo Leuzzi, Caterina Caputi, Filippo Manti, Stefano D’Arrigo, Arianna De Laurentiis, Claudio Graziano, Johannes R. Lemke, Federica Morelli, Danijela Petković Ramadža, Fabio Sirchia, Elisa Giorgio, Enza Maria Valente

**Affiliations:** 1 Department of Molecular Medicine, University of Pavia, Pavia, Italy; 2 Neurogenetics Research Centre, IRCCS Mondino Foundation, Pavia, Italy; 3 IRCCS Stella Maris Foundation, Pisa, Italy; 4 Department of Clinical ad Experimental Medicine, University of Pisa, Pisa, Italy; 5 Research Unit of Neuromuscular and Neurodegenerative Disorders, IRCCS Bambino Gesù Pediatric Hospital, Rome, Italy; 6 University Children’s Hospital, Zürich, Switzerland; 7 Department of Brain and Behavioral Sciences, University of Pavia, Pavia, Italy; 8 Child Neurology and Psychiatry Unit, IRCCS Mondino Foundation, Pavia, Italy; 9 Department of Human Neuroscience, Unit of Child Neurology and Psychiatry, Sapienza University of Rome, Rome, Italy; 10 Developmental Age Rehabilitation Service, Trasimeno District, Magione (PG), Italy; 11 Department of Developmental Neurology, Fondazione IRCCS Istituto Neurologico Carlo Besta, Milan, Italy; 12 Medical Genetics Unit, MeLabeT Department, AUSL Romagna, Cesena, Italy; 13 Institute of Human Genetics, University of Leipzig, Leipzig, Germany; 14 Department of Psychiatry, Autism Spectrum Disorders and Related Conditions Service, Lausanne University Hospital (CHUV), Lausanne, Switzerland; 15 Department of Pediatrics, University Hospital Centre Zagreb and University of Zagreb School of Medicine, Zagreb, Croatia; 16 Medical Genetics Unit, IRCCS San Matteo Foundation, Pavia, Italy; 17 Present Address: Department of Brain and Behavioral Sciences, University of Pavia, Pavia, Italy

**Keywords:** Genetics research, Disease genetics

## Abstract

Joubert syndrome (JS) is a genetically heterogeneous neurodevelopmental ciliopathy. Despite exome sequencing (ES), several patients remain undiagnosed. This study aims to increase the diagnostic yield by uncovering cryptic variants through targeted ES reanalysis. We first focused on 26 patients in whom ES only disclosed heterozygous pathogenic coding variants in a JS gene. We reanalyzed raw ES data searching for copy number variants (CNVs) and intronic variants affecting splicing. We validated CNVs through real-time PCR or chromosomal microarray, and splicing variants through RT-PCR or minigenes. Cryptic variants were then searched in additional 44 ES-negative JS individuals. We identified cryptic “second hits” in 14 of 26 children (54%) and biallelic cryptic variants in 3 of 44 (7%), reaching a definite diagnosis in 17 of 70 (overall diagnostic gain 24%). We show that CNVs and intronic splicing variants are a common mutational mechanism in JS; more importantly, we demonstrate that a significant proportion of such variants can be disclosed simply through a focused reanalysis of available ES data, with a significantly increase of the diagnostic yield especially among patients previously found to carry heterozygous coding variants in the *KIAA0586*, *CC2D2A* and *CPLANE1* genes.

## Introduction

Joubert syndrome (JS) is a genetically heterogeneous neurodevelopmental ciliopathy, diagnosed by the presence of a highly peculiar cerebellar and brainstem malformation, known as the “molar tooth sign” (MTS). The disease manifests early in life with hypotonia, developmental delay, oculomotor abnormalities, and respiratory pattern defects. Later signs include intellectual disability of variable severity, ataxia, and possible involvement of other organs such as the retina, kidneys, liver and skeleton [1].

More than 40 genetic forms of JS have been described, all following autosomal recessive inheritance, with the exceptions of X-linked *OFD1* and dominant *SUFU* [2]. Yet, despite extensive sequencing of known genes through next-generation sequencing (NGS)-based targeted panels or exome sequencing (ES), distinct studies over the past few years have detected biallelic pathogenic variants in about 60–90% patients [3]. This leaves a substantial proportion of families without a definite diagnosis, hampering proper counseling, management, and access to prenatal and preimplantation diagnosis.

Among known genes, six “major genes” (*CPLANE1*, *CEP290*, *AHI1*, *CC2D2A*, *TMEM67*, and *KIAA0586*) account for most diagnosed cases, with the remaining genes representing rare or ultra-rare causes of JS [3]. The quest for novel genes is still open, but it is unlikely that these alone can explain all negative cases. Recent evidences increasingly show that hidden heritability can be at least partly explained by the occurrence of cryptic pathogenic variants in known genes [4]. The term “cryptic” refers to variants that are either not easily captured or even undetectable by conventional diagnostic NGS techniques and related bioinformatic pipelines, which are mainly designed to disclose single nucleotide variants (SNVs) and small indels within exons and canonical splice sites. Cryptic variants may include complex genomic rearrangements, intronic variants disrupting splicing, and variants in regulatory regions affecting gene expression [5]. Moreover, intragenic copy number variants (CNVs) involving exonic and/or intronic regions of a gene can also remain undetected by conventional diagnostic NGS algorithms, especially those adopted in the past years, likely contributing to the proportion of undiagnosed cases.

Cryptic variants in JS-related genes have occasionally been reported. A paradigmatic example is the serendipitous identification of the deep intronic c.2991+1655 A > G splicing variant in the *CEP290* gene, which is a frequent cause of isolated Leber Congenital Amaurosis and also occurs in JS [6]. Few other reports have described the occasional detection of large deletions or intronic variants in JS-related genes such as *KIAA0586*, *TMEM67*, *CPLANE1, TCTN2, CEP120, CEP290* and *OFD1*, which were validated by studies on patients’ RNA [7–13].

Currently, genome sequencing (GS) is the gold standard technique to identify cryptic variants, as it can sequence beyond coding regions, and is able to detect SNVs as well as CNVs and more complex rearrangements [14]. However, in many countries including Italy, GS has not yet entered routine diagnostics, due to the still elevated costs, high amount of data to be analyzed and stored, and difficulties in data interpretation.

In the present study we tackle the issue of missing heritability in JS, hypothesizing that: (1) a relevant proportion of JS patients lacking a genetic diagnosis could harbor cryptic variants in a known gene, and, (2) at least a subset of these variants could be easily and quickly identified through a focused reanalysis of available ES data. To address these hypotheses, we adopted a proof-of-principle strategy by focusing on a sub-cohort of patients in whom ES identified only a single heterozygous pathogenic variant in a JS-related gene.

## Patients and methods

### Patients’ selection

This project relied on a large cohort of over 550 JS probands of European descent (mainly Italian) recruited in the Valente Lab since 2003. This cohort underwent genetic testing over twenty years, including either direct Sanger sequencing of some JS genes, targeted sequencing of a large panel of ciliopathy genes (comprising the majority of JS known genes) or, more recently, ES. Ethical approval for genetic testing of JS patients is in place, and all families signed a written informed consent for genetic testing. Through these approaches, biallelic pathogenic SNVs were so far identified in 357 patients.

Among patients lacking a conclusive diagnosis, we focused on the 70 JS children who failed to receive a definite genetic diagnosis by ES. We did not include patients who remained undiagnosed by “old” targeted panel sequencing, as in our experience this approach featured incomplete coverage across coding sequences and a relatively low diagnostic accuracy. Notably, 26 out of 70 children (37%) carried a single heterozygous pathogenic variant in a known autosomal recessive JS gene, including either loss-of-function variants (frameshift, nonsense or variants affecting canonical splice sites) or missense variants classified as pathogenic or likely pathogenic according to ACMG criteria [15, 16]. No other pathogenic variants in causative or candidate genes were identified by ES in these cases.

These 26 patients were initially selected for this study, while the remaining 44 ES-negative children were analyzed in a second step. Patients with monoallelic or biallelic variants of unknown significance (VUS) identified after ES were considered as negative cases and included in this second step.

Of the 26 single heterozygous carriers, 22 had a pathogenic variant in a major JS gene. The commonest genes were *CC2D2A* (NM_001378615.1), with 8 patients carrying 7 truncating and one missense variant, and *CPLANE1* (NM_023073.4), with 6 patients all carrying truncating variants. Four patients harbored the same heterozygous frameshift variant in *KIAA0586* (NM_001244189.2), two carried heterozygous truncating variants in *CEP290* (NM_025114.4), while the remaining six patients carried each a deleterious variant in either the *AHI1* (NM_001134831.2), *TMEM67* (NM_153704.6), *INPP5E* (NM_019892.6), *CEP120* (NM_001375405.1), *PIBF1* (NM_006346.4) and *TCTN1* (NM_001082538.3) genes (Supplementary Table 1).

### Statistical comparison of LOF allele frequencies

We sought to compare the frequencies of single heterozygous loss of function (LOF) variants in our JS cohort (ES-JS database) versus non-JS cohorts, including our ES-non-JS database, the Network for Italian Genomes - Exomes from Italy (NIG-ExIT; http://nigdb.cineca.it, accessed on April 2024) and gnomAD (https://gnomad.broadinstitute.org, v.3.1.2) databases. To this aim, we employed different approaches due to the different nature of the databases. In our ES-JS (*n* = 218) and ES-nonJS (*n* = 4328) cohorts and in the NIG-ExIT database (*n* = 1686), we derived the frequencies of single heterozygous LOF variants by referring to the total number of sequenced alleles in each group. Differently, in the gnomAD database, since each nucleotide is sequenced in a variable number of individuals, we calculated the frequencies of heterozygous LOF variants by referring to the weighted average of the times each single nucleotide harboring a LOF was sequenced in gnomAD, according to the following formula:

$${F}_{{global}}={\sum}_{i=1}^{m}\left(\frac{{n}_{i}}{{\sum }_{j=1}^{m}{n}_{j}}\times {f}_{i}\right)$$

Where:

- *m* is the total number of LOF alleles,
- *n*_*i*_ is the “Allele Number” for the LOF i-th allele,
- *f*_*i*_ is the frequency of the LOF i-th allele,
- *F*_*global*_ is the final “weighted” global frequency of all LOF alleles for each gene.

After this process, the weighted frequencies of LOF alleles for the considered genes in gnomAD were statistically compared to the frequencies of single heterozygous LOF variants in our JS cohort using a chi-square test.

### Identification of copy number variants

To search for in trans deletions or duplications, we exploited ExomeDepth [17] and CNVkit [18] algorithms along with a direct inspection of BAM files. Identified CNVs were verified either by quantitative real-time PCR (qRT-PCR) or chromosomal microarray analysis (Agilent Human Genome CGH Microarray Kit 4 × 180 k, Santa Clara, California, USA) on genomic DNA. Primers for qRT-PCR were designed within genomic regions flanking the predicted CNVs and are reported in Supplementary Table 2.

### Identification of intronic variants

To search for intronic variants possibly affecting splicing, we reanalyzed raw ES data from FASTQ files to include deep intronic variants, since conventional ES secondary analysis is usually set to filter intronic variants lying beyond 20–30 nucleotides from the exon-intron junction. In particular, we extended the .bed file (Human Core Exome, Twist Bioscience, San Francisco, California, USA) target regions by adding 200 nucleotides upstream and downstream each interval, to retain as many intronic variants as possible, even if poorly covered. We then inspected the genes of interest, focusing on intronic variants which were either absent or had a frequency lower than 0.5% both in gnomAD and in our internal ES-non-JS database. Retained variants were tested in silico for their potential ability to affect splicing using an array of informatic tools including Human Splicing Finder [19], SpliceAI [20] and Pangolin [21]. Variants fulfilling the above criteria and predicted to potentially affect splicing were confirmed by Sanger sequencing, and their correct segregation (i.e., in trans with the known pathogenic coding variant) was verified by sequencing the parents.

### Validation of the impact of intronic variants on splicing

To confirm the predicted impact of intronic variants on splicing, we followed two alternative strategies. In three patients, a fresh blood sample could be obtained. RNA was extracted from Tempus Blood RNA tubes (Applied Biosystem, Waltham, Massachusetts, USA) and then retrotranscribed to cDNA using the High-Capacity RNA-to-cDNA kit (Thermo Fisher Scientific, Waltham, Massachusetts, USA). A PCR was set up with specific primers designed within flanking exons (Supplementary Table 3), to amplify the region of interest. The PCR product was run on an agarose gel to detect the presence of two distinct bands. Individual bands were gel-excised and Sanger sequenced using the Big Dye Terminator chemistry (Thermo Fisher Scientific).

For the remaining patients, it was not possible to obtain a fresh sample for RNA extraction. To functionally validate these variants, minigene experiments were set up, as previously described [22]. Briefly, each genomic region containing the candidate splicing variant and flanking exons was PCR-amplified. The PCR product was cloned into a pGEM®-T Easy Vector (Promega, Madison, Wisconsin, USA) and transformed in the One-Shot TM TOP10 Chemically Competent E. coli (Invitrogen, Waltham, Massachusetts, USA) competent bacterial cells, following manufacturer’s protocol. Plasmids containing the wild-type or the mutated sequence were extracted using PureYield™ Plasmid Miniprep System (Promega) and then sub-cloned into pSPL3 vector (Life Sciences-Invitrogen, Waltham, Massachusetts, USA). The final pSPL3 plasmids containing the wild-type or the mutated sequence were transfected in HEK293-T cells using Lipofectamine 2000 (Thermo Fisher Scientific). After 24 h, RNA was extracted, retrotranscribed, a PCR was performed on cDNA using plasmid-specific primers and run on agarose gel as described above. Individual bands were excised and Sanger sequenced to assess the splicing defect at the RNA level. All cryptic variants confirmed to affect splicing were submitted to the Leiden Open Variation Database (LOVD) with the following individual accession numbers: #00449684, #00449685, #00449685, #00449687, #00449688, #00449689 and #00449690.

## Results

### Single heterozygous deleterious variants are strongly enriched in the JS cohort compared to control populations

For this “proof-of-principle” study, we initially focused on 26 JS individuals in whom ES had detected a single heterozygous pathogenic coding variant in a JS gene, as we reasoned that these patients had the highest chances to carry a cryptic variant in trans on the same gene.

To reinforce this hypothesis against the possibility that such single heterozygous variants could represent coincidental findings, we first compared the frequencies of heterozygous LOF alleles in five major genes (*CPLANE1*, *CEP290*, *AHI1*, *CC2D2A*, and *KIAA0586*) in our JS cohort with their respective frequencies in the Caucasian non-Finnish control population in gnomAD. We focused on LOF variants only, as the pathogenicity of missense variants would be much more difficult to assess. We also excluded *TMEM67*, since most variants found in this gene in JS patients are hypomorphic missense variants.

**Fig. 1:**
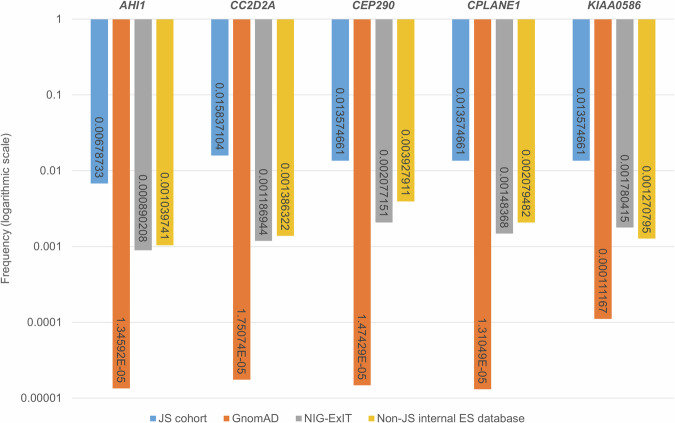
Comparison of frequencies of LOF alleles in five JS major genes showing a strong enrichment in the JS cohort (blue) compared to gnomAD population database (orange), NIG-ExIT database (gray) and non-JS internal ES database (yellow). The y-axis shows the frequencies expressed on a logarithmic scale.

The frequency of single heterozygous LOF variants in our JS cohort ranged between 0.7% and 1.4%, while these variants were extremely rare in gnomAD, with frequencies ranging from 0.001 to 0.01%. For each gene, such difference was strongly statistically significant (*p* < 0.00001) (Fig. 1). Similarly, single heterozygous LOF variants in the tested genes were significantly less common in our internal diagnostic database after excluding JS cases (0.10–0.39%), as well as in the NIG-ExIT database, containing aggregated data from 1686 healthy subjects of Italian origin (0.09–0.21%). These results support the hypothesis that the heterozygous pathogenic variants detected in JS patients do not represent a coincidental occurrence reflecting a mere “carrier status” for a recessive condition, but are likely causative of the disease, in trans with a second cryptic variant.

### Bioinformatic reanalysis of ES data disclosed potential cryptic “second hits” in over half patients

Since CNVs were not routinely detected in older bioinformatic pipelines, these variants may contribute to hidden heritability. Thus, we first searched for CNVs by combining bioinformatic tools with a manual re-inspection of BAM files focusing on the genes of interest, and detected large intragenic CNVs in six patients (Table 1). Four unrelated patients carried the same ~7 Kb intragenic deletion encompassing exons 8 to 10 in the *KIAA0586* gene, already reported in literature [23–25]. The remaining two patients carried an already reported deletion of ~5 Kb, encompassing exons 18 to 20 of the *AHI1* gene [26], and a duplication of ~88 Kb involving exons 17 to 49 of the *CPLANE1* gene, respectively. All CNVs were confirmed by quantitative real time PCR or chromosomal microarray analysis on genomic DNA and were shown to be in trans with the coding variant. Patients’ RNA was not available to assess the impact of these CNVs at the transcriptional level. No additional CNVs were detected in the remaining patients.

**Table 1:** CNVs detected upon ES reanalysis.

Family	Gene	Heterozygous coding variant	Second hit - CNV	ACMG – ClinGen CNV classification
COR62	*KIAA0586*	c.428del (p.Arg143LysfsTer4)	exon8_10 del	Likely pathogenic (1A-0; 2E-0.9; 3A-0)
COR164	*KIAA0586*	c.428del (p.Arg143LysfsTer4)	exon8_10 del	Likely pathogenic (1A-0; 2E-0.9; 3A-0)
COR93	*KIAA0586*	c.428del (p.Arg143LysfsTer4)	exon8_10 del	Likely pathogenic (1A-0; 2E-0.9; 3A-0)
COR130	*KIAA0586*	c.428del (p.Arg143LysfsTer4)	exon8_10 del	Likely pathogenic (1A-0; 2E-0.9; 3A-0)
COR127	*AHI1*	c.1500 C > G (p.Tyr500Ter)	exon18_20 del	Likely pathogenic (1A-0; 2E-0.9; 3A-0)
COR508	*CPLANE1*	c.3676 C > T (p.Arg1226Ter)	exon17_49 dup	Likely pathogenic (1A-0; 2I-0.9; 3A-0)

Next, we reanalyzed ES data searching for rare intronic variants potentially affecting splicing and identified candidate variants in nine patients, including three variants in *CPLANE1* (one recurrent in two unrelated patients), four in *CC2D2A* and one in *CEP290* (Table 2). Three variants (in the *CPLANE1* and *CC2D2A* genes) were within 20 bp from the exon-intron junction, but had not been reported in the former diagnostic report as they did not affect canonical splice sites. The remaining variants were deeper in the introns (from −23 up to +187) and had a coverage ranging from only 5 up to 124 reads. All variants were confirmed by Sanger sequencing and were demonstrated to be in trans with the coding variant.

**Table 2:** Intronic splicing variants detected upon ES reanalysis.

Family	Gene	Heterozygous coding variant	Second hit – predicted intronic splicing variant	Effect on splicing	Effect on protein	ACMG classification
COR528	*CC2D2A*	c.3084del (p.Lys1029ArgfsTer3)	c.3015-12 T > G	New acceptor splice site	p.Ile1006_Ser1010del5	Pathogenic (PM2; PP3; PS3; PM3; PM4)
COR19	*CC2D2A*	c.3084del (p.Lys1029ArgfsTer3)	c.1360-29 C > G	Alteration of branch point	p.Leu454ThrfsTer7	Pathogenic (PVS1; PM2; PS3; PM3)
COR152	*CC2D2A*	c.3289del (p.Val1097PhefsTer2)	c.2004-17 A > G	New acceptor splice site	p.Arg668SerfsTer68	Pathogenic (PVS1; PM2; PS3; PM3)
COR422	*CC2D2A*	c.3084del (p.Lys1029ArgfsTer3)	c.4315-23 T > C	Alteration of branch point	p.Ile1439_Gln1479del41	Pathogenic (PM3; PM2; PS3; PM4)
COR408	*CPLANE1*	c.8406del (p.Pro2804LeufsTer22)	c.8471-4_8471-3del	Alteration of acceptor splice site	p.Gly2824ValfsTer9	Pathogenic (PVS1; PM2; PS3; PM3)
COR590	*CPLANE1*	c.6700 C > T (p.Gln2234Ter)	c.2747-161 A > G	New acceptor splice site	p.Gly916Gly_Ala917ins21	Pathogenic (PM3; PM2; PS3; PM4)
COR445	*CPLANE1*	c.493del (p.Ile165AsnfsTer17)	c.1121+187 A > G	Alteration of SRE (ESE/ESS ratio)	p.Arg374SerfsTer10	Pathogenic (PVS1; PM2; PM3; PP3; PS3)
COR82	*CPLANE1*	c.4942_4945del (p.Ser1648HisfsTer26)	c.2747-161 A > G	New acceptor splice site	p.Gly916Gly_Ala917ins21	Pathogenic (PM3; PM2; PS3; PM4)
COR37	*CEP290*	c.5649dup (p.Leu1884ThrfsTer23)	c.7130-160 T > G	*New donor splice site (predicted but not validated)*	*-*	-

No additional cryptic variants were detected in the remaining 11 patients (Supplementary Table 4).

### Functional studies confirmed defective splicing for all but one candidate variants

**Fig. 2:**
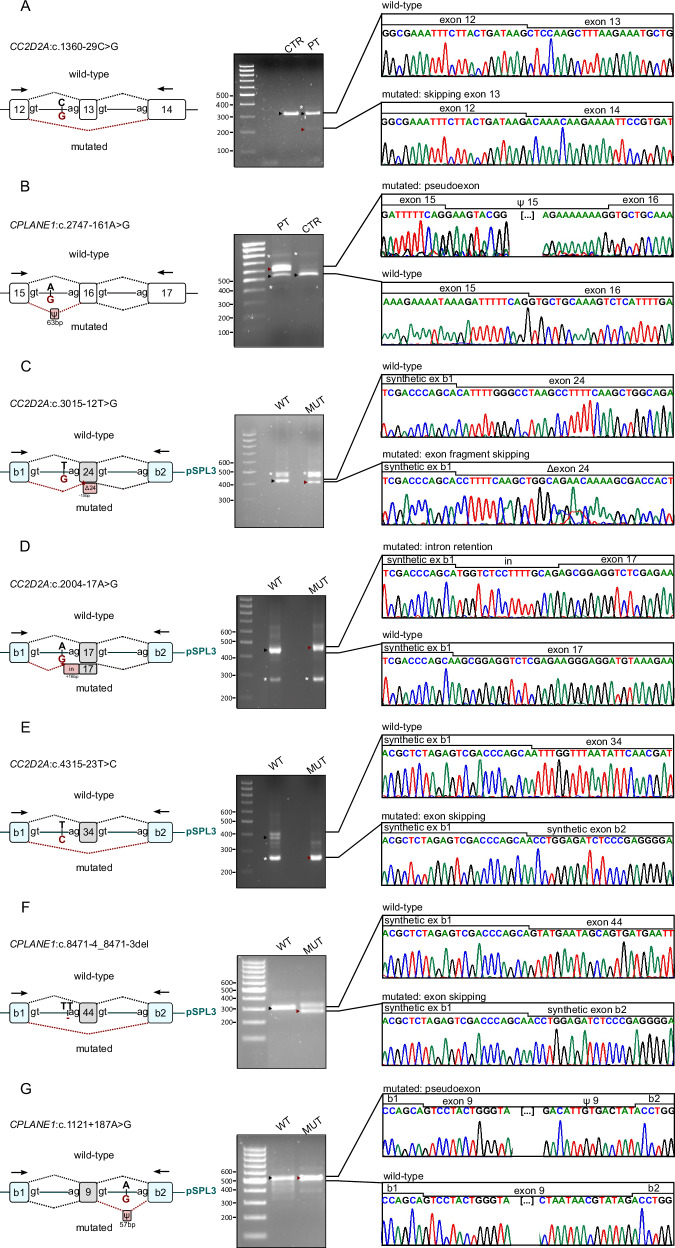
For each experiment, schematic splicing alterations (left), agarose gel electrophoresis on cDNA (middle) and electropherograms (right) are reported. **A** Direct RNA assay, the variant causes skipping of exon 13; (**B**) Direct RNA assay, a 63 bp pseudo-exon is inserted between exons 15 and 16; (**C**) Minigene, the variant disrupts the acceptor splicing site of exon 24, causing the loss of 15 bp; (**D**) Minigene, the variant introduces a new acceptor splicing site upstream exon 17, causing a 16 bp intron retention; (**E**, **F**) Minigenes, the variants cause the skipping of exons 34 and 44, respectively; (**G**) Minigene, a 57 bp pseudo-exon is inserted between exons 9 and 10. CTR control, PT patient, WT wild-type, MUT mutated, b1, b2: pSPL3 synthetic exons; black pins: wild-type bands; red pins: mutated bands; white asterisks: unexpected bands probably due to an internal splice site present in both the wild-type and mutated constructs in minigenes experiments.

Despite none of the intronic variants affected canonical donor or acceptor splice sites, they were predicted to alter splicing by in silico analyses, either by creating new acceptor splice sites, altering the branching points, or dysregulating splicing regulatory elements (Supplementary Table 5). We sought to validate these predictions by direct amplification and sequencing of patients’ RNA or, in the absence of such biological material, by the development of customized minigenes. Schematic results of in vitro assays are summarized in Fig. 2, while the resulting effect on proteins is reported in Table 2.

Notably, we were able to confirm a defective splicing for all tested variants but one, the *CEP290* c.7130-160 T > G variant, for which both cDNA sequencing and minigene assay failed to detect overt splicing defects (Supplementary Fig. 1).

Variants c.1360-29 C > G in the *CC2D2A* gene and c.2747-161 A > G in the *CPLANE1* gene were evaluated directly by sequencing patients’ RNA samples. The first variant was shown to cause skipping of exon 13, resulting in a prematurely truncated protein, while the second (recurring in three unrelated patients) introduced a 63 bp pseudo-exon *in frame* between exons 15 and 16, and is therefore expected to include 21 additional amino acids in the protein.

For all remaining variants, through the development of minigene plasmids, we were able to demonstrate the activation of new acceptor splice sites (for *CC2D2A* c.3015-12 T > G and *CC2D2A* c.2004-17 A > G), exon skipping (for *CC2D2A* c.4315-23 T > C and *CPLANE1* c.8471-4_8471-3del) or the introduction of a pseudo-exon (for *CPLANE1* c.1121+187 A > G). At the protein level, all these splicing defects resulted in premature protein truncation or, in the case of *CC2D2A* c.4315-23 T > C and *CC2D2A* c.3015-12 T > G, the *in frame* deletion of 41 and 5 amino acids, respectively.

### Reanalysis of patients with negative ES

Lastly, we performed bioinformatic reanalysis of ES data in the remaining 44 negative patients and identified biallelic cryptic variants in three additional cases (Table 3). Two patients were homozygous for the *CPLANE1* c.8471-4_8471-3del variant, while the third one carried the *CPLANE1* c.2747-161 A > G deep intronic variant in the heterozygous state. In this patient, reanalysis further disclosed a heterozygous *in frame* deletion spanning ~1Kb, encompassing exons 7 and 8 of the *CPLANE1* gene, in trans with the deep intronic variant.

**Table 3:** Biallelic cryptic variants detected in ES-negative patients.

Family	Gene	Variant 1	Variant 2
COR99	*CPLANE1*	c.2747-161 A > G (p.Gly916Gly_Ala917ins21)	exon7_8del
COR437	*CPLANE1*	[c.8471-4_8471-3del (p.Gly2824ValfsTer9); c.8471-4_8471-3del (p.Gly2824ValfsTer9)]	
COR545	*CPLANE1*	[c.8471-4_8471-3del (p.Gly2824ValfsTer9); c.8471-4_8471-3del (p.Gly2824ValfsTer9)]	

## Discussion

Here we demonstrate that CNVs and intronic variants affecting splicing are common mutational mechanisms in JS, and that at least a proportion of these variants can be safely identified through a focused reanalysis of available ES data. This approach, easily exportable to the diagnostic setting, can significantly increase the diagnostic yield of JS, providing substantial benefits for patients and families. A timely diagnosis of the underlying genetic defect would allow families to receive adequate genetic counseling for future pregnancies and gain access to prenatal and preimplantation diagnosis. Even more importantly, a genetic diagnosis is pivotal to provide affected children with a correct prognosis and establish the most appropriate management plan for prevention and treatment of potential complications. For instance, renal complications such as nephronophthisis, occurring in about one third of JS patients, usually present insidiously towards the end of the first decade and are potentially life-threatening in the absence of specific management and treatment. While clinical and laboratory indicators of renal involvement may not be clearly detectable at onset, the early identification of pathogenic variants in genes carrying a strong risk of renal involvement (such as *CEP290*) allow for the appropriate surveillance for renal issues since the first years of life, while mutations in genes that rarely associate to kidney defects (e.g., *CPLANE1*) can reassure families on a very low risk of renal failure [1, 2].

In the pre-NGS era, the genetic diagnosis of JS was extremely difficult due to the high number of disease-causing genes, many of which consisting of a large number of exons. The widespread adoption of NGS greatly improved the diagnostic rate in JS, yet a variable proportion of patients (10 to 35% in different studies) still remains undiagnosed [3]. Recent evidence suggests that cryptic pathogenic variants, which are not easily detectable with conventional diagnostic routine, may account for a relevant subset of allegedly “negative” cases, both in JS as well as in other inherited rare diseases [7–13]. Indeed, it has been reckoned that between 9 and 30% of causative variants in patients with rare genetic disorders might act through disruption of splicing [27], yet only 8.6% (24,976/289,000) of all variants reported in the Human Gene Mutation Database (HGMD) are splicing mutations [28], suggesting they may be extensively underestimated. In this line, a recent study performed deep targeted sequencing of the genomic region encompassing the *ABCA4* gene in a cohort of 67 patients with retinal dystrophy carrying single coding pathogenetic variants, leading to the identification of nine distinct cryptic variants in 21 probands, with a diagnostic gain of 31% [29].

In the present study, ES reanalysis in 26 JS patients heterozygous for a pathogenic coding variant led to a diagnostic gain of 54% (14 out of 26), including intragenic CNVs in 6 patients (23%) and cryptic variants affecting splicing in 8 (31%). When considering the whole cohort of 70 ES-negative cases, a definite diagnosis was reached in 17 patients, resulting in an overall diagnostic yield of 24%. Notably, the vast majority of detected variants fell within three major JS-genes, namely *KIAA0586*, *CC2D2A* and *CPLANE1*, and some of them recurred in multiple unrelated patients, suggesting a founder effect [25]. It is worth noting that the identification of all variants reported in this study was obtained only through a reanalysis of available ES data, without performing additional wet-lab experiments, such as GS or RNA sequencing. This shows that a focused implementation of bioinformatic pipelines may represent a powerful approach adoptable in the diagnostic setting to disclose not only CNVs but also a subset of intronic variants, which will likely significantly increase the diagnostic yield, especially when a single pathogenic coding variant had already been identified. This could be particularly relevant for variants affecting splicing, for which targeted therapeutic strategies based on antisense oligonucleotides are being developed to effectively revert the splicing defect, at least in accessible tissues such as the retina. It is clearly emerging how this approach can hold a great potential for treating retinal dystrophy, a severe condition which occurs in about one third of JS patients. Several clinical trials have given promising results [30], among which the trial with the splicing-modulating antisense oligonucleotide Sepofarsen, which targets the common intronic splice-site variant c.2991+1655 A > G in the *CEP290* gene [31, 32].

We acknowledge the fact that certain variants, such as the intragenic CNVs and the *CPLANE1* c.8471-4_8471-3del splicing variant, should not be considered “cryptic” anymore, as they would have been detected in current diagnostic settings using up-to-date bioinformatics pipelines. Algorithms to detect CNVs from ES data were only recently implemented in most diagnostic labs, thanks to the development of some user-friendly bioinformatics tools, resulting in a significant increase of the diagnostic yield for several rare genetic disorders [33]. However, even in a recent past such variants were regularly missed, as appropriate CNV callers were lacking. Also the *CPLANE1* splicing variant c.8471-4_8471-3del was not detected in the initial ES analysis, likely due to early limitations in correctly classifying splicing variants outside the canonical donor and acceptor sites. Thus, besides disclosing “true cryptic” variants, a targeted reanalysis of ES data may also highlight variants which the original analysis failed to detect, due to its limited sensitivity and robustness in correctly detecting and classifying certain types of variants. Indeed, in our cohort all these variants were disclosed in exomes that had been formerly analyzed some years ago. This highlights the importance of periodically re-analyzing old negative ES using updated tools, as this simple and fast strategy is likely to significantly reduce missing heritability, by detecting not only “true cryptic” variants, but also “former cryptic” variants missed by previous analyses. Similarly to coding variants, both CNVs and intronic variants also require validation through alternative techniques. While CNVs can be easily validated by real time PCR on genomic DNA, demonstration of the effect of putative splicing variants at the RNA level is crucial and requires RNA obtained from fresh blood or cell cultures. As obtaining these additional samples is not always possible, we confirm the minigene technique as a reliable, cost-effective, and relatively fast alternative strategy to validate the effect of splicing variants, in line with former evidences from the literature [34, 35]. In the present study, only one out of eight candidate intronic variants predicted to affect splicing could not be validated as pathogenic. This high success rate confirms the reliability of the proposed approach to identify a substantial subset of cryptic variants, suggesting this strategy can also be adopted for other rare disorders with hidden heritability.

Alternative strategies will be required to solve the remaining negative cases, whose second hits may be represented by deeper intronic variants or by structural variants affecting regulatory elements or disrupting topologically associated domains, which are truly undetectable by ES and whose disclosure requires distinct approaches such as GS, RNA sequencing, Hi-C, Optical Genome Mapping or long-read sequencing [36]. Another relevant contribution towards an increase in the diagnostic yield will likely come from improvements in the classification and functional validation of variants of unknown significance, especially missense hypomorphic variants, which we and others showed to occur in JS and other ciliopathies, and to bear a pathogenic impact despite low pathogenicity prediction scores [25, 37]. Finally, we would like to mention that negative genetic testing may also result from an incorrect recognition of the MTS, wrongly addressing patients affected by distinct rare neurodevelopmental disorders to JS gene panel testing [38]. Despite the significant progresses made by genetic technologies, a careful clinical assessment of patients still remains an essential step for a successful diagnostic process.

## Supplementary information

Supplementary material_clean

## Data Availability

The authors declare that all data generated or analyzed during this study and supporting the findings of the study are available within the paper and its supplementary information files.
