# Supplementary material for: Pathogenic cryptic variants detectable through exome data reanalysis significantly increase the diagnostic yield in Joubert syndrome
Source: Eur J Hum Genet. 2024 Oct 11;33(1):72–9. doi: 10.1038/s41431-024-01703-x (PMC11711660; doi:10.1038/s41431-024-01703-x)
Supplement: Supplementary file 1 — Supplementary material_clean [file 41431_2024_1703_MOESM1_ESM.pdf]

# Supplementary material

## Supplementary Table 1 – List of 26 patients carrying a monoallelic pathogenetic variant

| Family | Gene | Heterozygous coding variant | ACMG classification |
|---|---|---|---|
| COR178 | *CC2D2A* | c.3341C>T (p.Thr1114Met) | Pathogenic (PP5; PP3; PM1; PM2) |
| COR353 | *CC2D2A* | c.2848C>T (p.Arg950Ter) | Pathogenic (PVS1; PS3; PP5; PM2) |
| COR430 | *CC2D2A* | c.523del (p.Ile175SerfsTer83) | Pathogenic (PVS1; PP5; PM2) |
| COR433 | *CC2D2A* | c.384del (p.Arg129AspfsTer23) | Pathogenic (PVS1; PP5; PM2) |
| COR528 | *CC2D2A* | c.3084del (p.Lys1029ArgfsTer3) | Pathogenic (PVS1; PP5; PM2) |
| COR19 | *CC2D2A* | c.3084del (p.Lys1029ArgfsTer3) | Pathogenic (PVS1; PP5; PM2) |
| COR152 | *CC2D2A* | c.3289del (p.Val1097PhefsTer2) | Pathogenic (PVS1; PP5; PM2; PS3) |
| COR422 | *CC2D2A* | c.3084del (p.Lys1029ArgfsTer3) | Pathogenic (PVS1; PP5; PM2) |
| COR200 | *CPLANE1* | c.8162_8163del (p.Ile2721ArgfsTer8) | Pathogenic (PVS1; PM2) |
| COR508 | *CPLANE1* | c.3676C>T (p.Arg1226Ter) | Pathogenic (PVS1; PP5; PM2) |
| COR408 | *CPLANE1* | c.8406del (p.Pro2804LeufsTer22) | Pathogenic (PVS1; PM2) |
| COR590 | *CPLANE1* | c.6700C>T (p.Gln2234Ter) | Pathogenic (PVS1; PP5; PM2) |
| COR445 | *CPLANE1* | c.493del (p.Ile165AsnfsTer17) | Pathogenic (PVS1; PP5; PM2) |
| COR82 | *CPLANE1* | c.4942_4945del (p.Ser1648HisfsTer26) | Pathogenic (PVS1; PM2) |
| COR62 | *KIAA0586* | c.428del (p.Arg143LysfsTer4) | Pathogenic (PVS1; PP5; PM2) |
| COR164 | *KIAA0586* | c.428del (p.Arg143LysfsTer4) | Pathogenic (PVS1; PP5; PM2) |
| COR93 | *KIAA0586* | c.428del (p.Arg143LysfsTer4) | Pathogenic (PVS1; PP5; PM2) |
| COR130 | *KIAA0586* | c.428del (p.Arg143LysfsTer4) | Pathogenic (PVS1; PP5; PM2) |
| COR522 | *CEP290* | c.6869del (p.Asn2290IlefsTer11) | Pathogenic (PVS1; PP5; PM2) |
| COR37 | *CEP290* | c.5649dup (p.Leu1884ThrfsTer23) | Pathogenic (PVS1; PP5; PM2) |
| COR127 | *AHI1* | c.1500C>G (p.Tyr500Ter) | Pathogenic (PVS1; PP5; PM2) |
| COR475 | *TMEM67* | c.641A>G (p.Tyr214Cys) | Likely pathogenic (PM1; PM2; PP3; PP4) |
| COR25 | *INPP5E* | c.1379C>G (p.Ser460Cys) | Likely pathogenic (PM2; PP2; PP3; PP4) |
| COR420 | *CEP120* | c.594del (p.Phe198LeufsTer8) | Pathogenic (PVS1; PM2) |
| COR536 | *PIBF1* | c.895C>T (p.Arg299Ter) | Pathogenic (PVS1; PM2) |
| COR39 | *TCTN1* | c.736A>T (p.Lys246Ter) | Pathogenic (PVS1; PP5; PM2) |

**Supplementary Table 2 – primers and real-time PCR conditions for validation of CNVs on genomic DNA**

| Primer | Sequence | Temperature of melting |
|---|---|---|
| KIAA0586_7F<br>KIAA0586_7R | GAAGATGCAGGCATAGAGAAGG<br>CCACGGTTGTAGCTGAATCAAT | 60°C |
| KIAA0586_8F<br>KIAA0586_8R | AGACTGATAAACACCTGCAACG<br>GTGCTGCTCCATAAACACATTC | 60°C |
| KIAA0586_10F<br>KIAA0586_10R | AAATCTCCTTTGGAGACACCAG<br>ATCCCTTGAAACAGGTACAGGA | 60°C |
| KIAA0586_11F<br>KIAA0586_11R | GAATCATCAAACACCACCTCAC<br>TGTTTTCTCAGGAGTCCATCC | 60°C |

**Supplementary Table 3 – primers and PCR conditions for validation of splicing effect at the RNA level**

| Primer | Sequence | Temperature of melting | Variant |
|---|---|---|---|
| CC2D2A_c12F<br>CC2D2A_c14R | TCACTCATCATCCCTGTTTTAGC<br>ACTCTCGAAGGGATTTCATCTC | 58°C | *CC2D2A:*<br>c.1360-29C>G |
| CPLANE1_c15F<br>CPLANE1_c17R | GGACGTATTTTCTTCAGATACGCT<br>AGCTGGAAAGCCACACCAAT | 59°C | *CPLANE1:*<br>c.2747-161A>G |
| CEP290_c52F<br>CEP290_c54R | TCAGCTGGATAAAGAGAAAGCAGA<br>TCAAACTCTTCAGAAGCAGCA | 59°C | *CEP290:*<br>c.7130-160T>G |

**Supplementary Table 4 – Patients with single heterozygous pathogenic SNVs negative at ES reanalysis**

| Family | Gene | Heterozygous coding variant |
|---|---|---|
| COR178 | *CC2D2A* | c.3341C>T (p.Thr1114Met) |
| COR353 | *CC2D2A* | c.2848C>T (p.Arg950Ter) |
| COR430 | *CC2D2A* | c.523del (p.Ile175SerfsTer83) |
| COR433 | *CC2D2A* | c.384del (p.Arg129AspfsTer23) |
| COR200 | *CPLANE1* | c.8162_8163del (p.Ile2721ArgfsTer8) |
| COR522 | *CEP290* | c.6869del (p.Asn2290IlefsTer11) |
| COR475 | *TMEM67* | c.641A>G (p.Tyr214Cys) |
| COR420 | *CEP120* | c.594del (p.Phe198LeufsTer8) |
| COR25 | *INPP5E* | c.1379C>G (p.Ser460Cys) |
| COR536 | *PIBF1* | c.895C>T (p.Arg299Ter) |
| COR39 | *TCTN1* | c.736A>T (p.Lys246Ter) |

**Supplementary Table 5 – gnomAD frequency and splicing predictions of identified intronic variants**

| Intronic variant | GnomAD frequency | HSF prediction | SpliceAI prediction | Pangolin prediction |
|---|---|---|---|---|
| *CC2D2A* c.3015-12T>G | 0% | Broken WT acceptor site | Acceptor loss (0.47) | Splice Loss (0.26) |
| *CC2D2A* c.1360-29C>G | 0% | Broken WT branch point | Acceptor loss (0.46) | Splice Loss (0.20) |
| *CC2D2A* c.2004-17A>G | 0% | New acceptor splice site | Acceptor loss (0.23) | Splice Loss (0.17) |
| *CC2D2A* c.4315-23T>C | 0.000436% | Broken WT branch point | Acceptor loss (0.60) | Splice Loss (0.37) |
| *CPLANE1* c.8471-4_8471-3del | 0.00297% | Broken WT acceptor site | Acceptor loss (0.58) | Splice Loss (0.46) |
| *CPLANE1* c.2747-161A>G | 0% | New acceptor splice site | Acceptor gain (0.72) | Splice Gain (0.77) |
| *CPLANE1* c.1121+187A>G | 0.00132% | Significant alteration of ESE/ESS motifs ratio (4) | Acceptor gain (0.67) | Splice Gain (0.30) |
| *CEP290* c.7130-160T>G | 0.0255% | New donor splice site | Acceptor loss (0.01) | Splice Loss (0.01) |

**Supplementary Figure 1 – CEP290: c.7130-160T>G minigene**

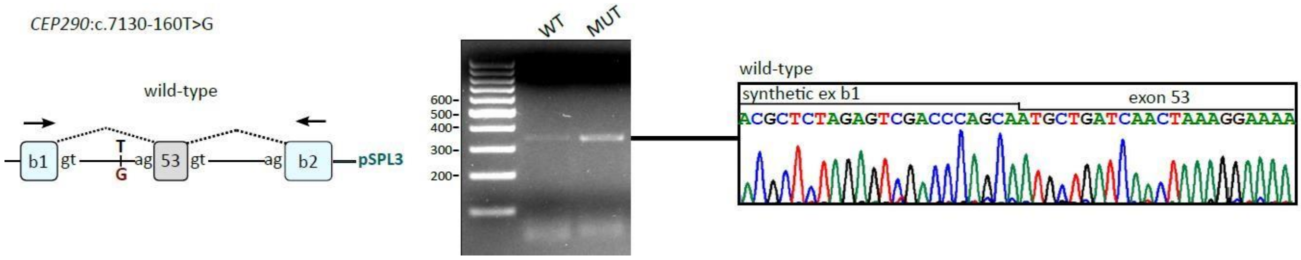


Schematic wild type splicing (left), agarose gel electrophoresis on cDNA (middle) and electropherograms (right) of negative minigene on *CEP290*: c.7130-160T>G variant are reported. Both constructs show the non-altered wild-type splicing of exon 53. WT: wild-type; MUT: mutated; b1, b2: pSPL3 synthetic exons.
